# Examining Pediatric Resident Electronic Health Records Use During Prerounding: Mixed Methods Observational Study

**DOI:** 10.2196/38079

**Published:** 2023-05-10

**Authors:** Jawad Alami, Clare Hammonds, Erin Hensien, Jenan Khraibani, Stephen Borowitz, Martha Hellems, Sara Riggs

**Affiliations:** 1 Department of Systems and Information Engineering University of Virginia Charlottesville, VA United States; 2 Department of Computer and Communication Engineering American University of Beirut Beirut Lebanon; 3 Department of Pediatrics University of Virginia Charlottesville, VA United States

**Keywords:** EHR, pediatric, usability, prerounding, training, electronic health record, eHealth

## Abstract

**Background:**

Electronic health records (EHRs) play a substantial role in modern health care, especially during prerounding, when residents gather patient information to inform daily care decisions of the care team. The effective use of the EHR system is crucial for efficient and frustration-free prerounding. Ideally, the system should be designed to support efficient user interactions by presenting data effectively and providing easy navigation between different pages. Additionally, training on the system should aim to make user interactions more efficient by familiarizing the users with best practices that minimize interaction time while using the full potential of the system’s capabilities. However, formal training on EHR systems often falls short of providing residents with all the necessary EHR-related skills, leading to the adoption of inefficient practices and the underuse of the system’s full range of capabilities.

**Objective:**

This study aims to examine the efficiency of EHR use during prerounding among pediatric residents, assess the effect of experience level on EHR use, and identify areas for improvement in EHR design and training.

**Methods:**

A mixed methods approach was used, involving a self-reported survey and video analysis of prerounding practices of the entire population of pediatric residents from a large teaching hospital in the South Atlantic Region. The residents were stratified by experience level by postgraduate year. Data were collected on the number of pages accessed, duration of prerounding, task completion rates, and effective use of data sources. Observational and qualitative data complemented the quantitative analysis. Our study followed the STROBE (Strengthening the Reporting of Observational Studies in Epidemiology) reporting guidelines, ensuring completeness and transparency of reporting.

**Results:**

Of the 30 pediatric residents, 20 were included in the analyses; of these, 16 (80%) missed at least 1 step during prerounding. Although more experienced residents on average omitted fewer steps, 4 (57%) of the 7 most experienced residents still omitted at least 1 step. On average, residents took 6.5 minutes to round each patient and accessed 21 pages within the EHR during prerounding; no statistically significant differences were observed between experience levels for prerounding times (*P*=.48) or number of pages accessed (*P*=.92). The use of aggregated data pages within the EHR system neither seem to improve prerounding times nor decrease the number of pages accessed.

**Conclusions:**

The findings suggest that EHR design should be improved to better support user needs, and hospitals should adopt more effective training programs to familiarize residents with the system’s capabilities. We recommend implementing prerounding checklists and providing ongoing EHR training programs for health care practitioners. Despite the generalizability of limitations of our study in terms of sample size and specialization, it offers valuable insights for future research to investigate the impact of EHR use on patient outcomes and satisfaction, as well as identify factors that contribute to efficient and effective EHR usage.

## Introduction

Over the past 2 decades, electronic health record (EHR) systems have increasingly been incorporated into the workflow of physicians and other clinicians in hospitals across the United States [1]. Although EHR systems have the potential to improve the quality of patient care and streamline health care workflows [2], in reality, clinicians have often reported negative impacts on patient care, job satisfaction, and increased burnout due to EHR system implementation and use [3-6]. Recent studies have estimated that physicians spend upward of two-thirds of their time documenting and reviewing patient encounters in the EHR and only one-third of their time providing direct care to patients [2]. For over a decade, EHR systems’ usability issues [7-9] and best practices for better implementation [5] have been identified; despite that, overall satisfaction with EHR use has not improved [10,11], and the EHR system continues to have negative effects on workflow and patient care [12,13].

Prerounding an inpatient is an information retrieval task that relies heavily on the EHR system. In a teaching hospital, resident physicians review their patients’ records during prerounding to (1) form a mental model about the patient’s medical history, recent events, and current status and (2) then, communicate this information to the entire care team during rounds. This is especially critical in pediatrics as multiple stakeholders are involved with the patient care (ie, clinicians, nurses, specialists, and caregivers), the data collected during prerounding can directly affect the outcome of family-centered rounding [14].

During this process, residents access numerous sections in the EHR system to retrieve information that is documented in various locations and formats; additionally, they are often under time pressure as they must collect and compile patient information at the start of their shifts to present a case summary to the care team during rounds.

Residents usually receive some formal training on EHR usage; however, concerns about the quality and depth of training have been expressed throughout the literature [15-18]. EHR training is typically generic and not workflow-specific [7], leaving residents unaware of all the EHR functionalities that could improve the prerounding process and workflow [19-22]. Instead of relying on systemic training, residents typically learn EHR “best practices” informally from other more experienced residents and attending physicians. This often leads them to adopt strategies that they have observed or that were passed down through word of mouth [23,24].

Furthermore, evidence suggests that EHR usage among residents is neither effective nor efficient. Residents spend more than 40% of their time interacting with the EHR, making up to 4000 clicks per shift [25,26], but clinicians still omit recording 22% and verbalizing 42% of patient data from intensive care unit (ICU) rounds presentations [27]. Inadequate EHR training has been linked to clinician frustration, inefficiency, and medical errors, even among clinical experts [28,29]. Despite the large amount of time clinicians spend using the EHR, a large survey from American EHR Partners found that almost half of the clinicians surveyed had no more than 3 days of training on the EHR system they use [30]. According to EHR providers, the current training process is inadequate in medical institutions [30,31]. The American Medical Association [32] compares EHR training sessions to having

an architecture student...only receiving minimal instruction on computer aided design (CAD) programs; then, being expected to expertly use CAD to its full potential on a daily basis once out in the workplace.

In this mixed methods observational study, we aim to investigate how first-, second-, and third-year pediatric residents in the Acute Care Wards, who have not received any formal training on prerounding, use an EHR system. We explore the perceptions of their own performance and how it relates to their actual performance, and determine whether their performance improves with more experience and exposure. Despite the lack of formal training, we expect more experienced residents to be more efficient in prerounding.

Our study seeks to identify potential areas for improvement and inform the design of training programs to reduce errors, increase efficiency, and enhance resident satisfaction. By comparing our findings with previous studies examining prerounding in various specialties, we aim to identify emerging patterns and guide the development of training practices and design solutions that could enhance residents’ EHR interactions and improve patient care.

## Methods

### Study Design

This study was designed as a mixed methods approach combining quantitative and qualitative analyses to evaluate residents’ prerounding performance using the Epic EHR system. We invited pediatric residents at a large teaching hospital in the South Atlantic Region to participate in the study as part of an optional professional development event. A convenience sample of all 30 pediatric residents voluntarily participated are reflecting the entire population of pediatric residents in the hospital. The residents’ level of training ranged from 1 to 3 years of postgraduate medical education, and all residents had more than a month of direct patient care in the pediatric wards. All residents had prior experience using the EHR system (Epic Systems) for prerounding as part of their work routine. To ensure completeness and transparency of reporting, we followed the STROBE (Strengthening the Reporting of Observational Studies in Epidemiology) reporting guidelines [33].

### Data Collection

Several days prior to the professional development event, participants were asked to complete a web-based questionnaire asking about their prerounding experience. At the start of the professional development event, participants were also requested to complete another demographic and EHR usability questionnaire (see Multimedia Appendix 1 for more details on the questionnaires).

For the experimental portion of the study, residents were instructed to perform their prerounding routine on 2 pediatric inpatients. All residents who participated in this study prerounded on the same 2 patients. Both cases were of medium complexity and representative of the types of patients that residents routinely care for in the acute care wards (for more details, see Multimedia Appendix 2).

Each resident was provided a 17.3″ Lenovo workstation laptop with a wireless mouse that had Epic EHR system preinstalled. Upon logging in the system, the workstation displayed the same EHR layout that the residents typically use to preround with any customizations of the EHR system they have created. Morae video analysis software was also installed to record residents’ speech and video capture all user interactions with the system. Residents were also provided paper so that they could write down any information they normally write down during prerounding to serve as their notes during rounds. Residents were seated in proximity to each other, similar to the environment in which residents typically preround in.

At the beginning of the study, residents were given the names of the 2 case-study patients and were asked to log into their accounts in the EHR system and initiate the video-capturing software. The video-capturing software would then prompt the residents to complete a small questionnaire related to their experience and the EHR system’s usability. After completing the questionnaire, residents began prerounding on the 2 case-study patients using the think-aloud protocol to verbalize their internal thought processes while completing the tasks.

The study involved 2 groups of 15 residents who alternated prerounding on the patients. Each group was allotted a maximum of 20 minutes to complete prerounding on both patients. This time limit was determined by pediatric experts, based on the relative complexity of the cases and questionnaire responses, where the majority of residents indicated that they usually need less than 10 minutes for prerounding a patient. After residents prerounded both patients, they were asked to fill out a debriefing questionnaire on their experience during the study, their concerns about the time constraints, and any difficulty they encountered while completing the prerounding tasks.

### Data Analysis

A team of 5 researchers used a standardized spreadsheet to systematically categorize the data collected from the Morae video analysis software during the recordings of the prerounding process. To ensure consistency in video analysis, prerounding data collection was categorized into the following six tasks based on literature [34-36] and recommendations of pediatric experts who assisted in conducting the study. These tasks included (1) reviewing patient vital signs (vitals), (2) checking prior and upcoming feeding and lab orders (orders), (3) reviewing recent lab results (labs), (4) checking patient intakes and outputs (IOs), (5) reviewing clinicians’ and nurses’ notes (notes), and (6) reviewing current medications and medication changes (meds). These tasks served as a benchmark for evaluating residents’ performance, as they are expected to complete all 6 tasks for each patient. We analyzed the video recordings to determine whether each task was completed or omitted, the time taken to complete each task, and any participant comments related to the task being performed, including any difficulties or challenges encountered. To facilitate the analysis process, standardized drop-down menus were used to populate the spreadsheet with 5 events, including the start or end of prerounding of the patient, start or end of a task, page access, information or data collection, and participant comments. The video reviewer created an entry for each event by recording the timestamp of the event and using the drop-down menu to populate the entry with the relevant event type, prerounding task being performed, task, and the page being viewed, alongside any comments made by the resident (see Multimedia Appendix 3).

To ensure the reliability of our data, we used a rigorous 2-reviewer approach, where each video recording was independently analyzed and coded. The level of agreement among reviewers was very high, with less than 5% (80/1926) of entries showing discrepancies between reviewers. A third reviewer was assigned to reconcile any discrepancies and consolidate similar entries, and all proposed changes or modifications were mutually agreed upon by all reviewers before proceeding to the analysis phase of the study.

### Outcome Variables

To assess the effectiveness of the prerounding process, several outcome variables were analyzed:

- Task omission rates: Task omission rates were calculated as the percentage of residents who omitted each task for 1 or both patients and the percentage of residents who omitted at least 1 task, categorized by experience level.
- Number of pages accessed: The number of distinct pages accessed during prerounding and the mean number of pages accessed by residents when prerounding a patient, categorized by experience level were recorded.
- Prerounding duration: Prerounding duration for each patient was categorized and analyzed by experience level.
- Use of aggregated data pages: The use of aggregated data pages was analyzed, including the mean number of pages accessed and prerounding duration for residents who used these pages, and how their use impacted performance.

These outcome variables provide valuable insights into the effectiveness of the prerounding process and the performance of residents.

### Statistical Analysis

We performed statistical analysis using Excel (Microsoft Corporation) for data entry and SPSS (IBM Corp) for data analysis. Categorical variables were presented as frequencies and percentages. To investigate the association between variables, we used the independent sample *t* test and ANOVA. A *P* value less than .05 was considered statistically significant.

### Ethics Approval

Ethics approval for this study was obtained from the institutional review board for Social and Behavioral Sciences (IRB-SBS) at the University of Virginia (IRB protocol number is 3480). All participants provided informed consent before taking part in the study.

### Funding

This study had no external funding to declare. All aspects of the research, including design, data collection, analysis, and publication, were independently managed by the authors.

## Results

### Participant Demographics

A total of 30 pediatric residents participated in our study, but due to technical issues related to data extraction (specifically, corrupted recording files), only 20 residents (16 females and 4 males) had video recordings that could be analyzed. The analyzed video recordings were evenly distributed across residents of different pediatric department experience levels, with 7 PGY-1 (postgraduate year) residents, 6 PGY-2 residents, and 7 PGY-3 residents.

### Data Omission

Based on the debriefing survey presented at the conclusion of the study, only 2 residents (10%) reported not having enough time to preround, and only 1 participant (5%) reported not being able to find all the information they searched for. However, based on the video analysis we found that 16 residents (80%) did not complete at least 1 task. Table 1 shows the tasks that were omitted and whether they were omitted for 1 or both patients. The task “meds” (ie, reviewing medications and medication changes) was the most overlooked task; 7 residents omitted the task for both patients, and 4 residents omitted it for 1 patient. For the task “orders” (ie, reviewing feeding and laboratory orders), 5 residents omitted this task for both patients, and another 5 residents omitted it for 1 patient. Finally, only 1 participant omitted checking “IOs” (ie, checking intakes and outputs) for 1 patient. The 3 remaining tasks—that is, “labs,” “notes,” and “vitals”—were completed by all residents.

**Table 1:** Number of residents (N=20) who omitted each task for 1 or both patients.

Task	Participants who omitted a task, n (%)		
	For both patients	For 1 patient	For at least 1 patient
Meds	7 (35)	4 (20)	11 (55)
Orders	5 (25)	5 (25)	10 (50)
IOs^a^	0 (0)	1 (5)	1 (5)
Labs	0 (0)	0 (0)	0 (0)
Notes	0 (0)	0 (0)	0 (0)
Vitals	0 (0)	0 (0)	0 (0)
Total	12 (60)	9 (45)	16 (80)

^a^IO: intake and output.

We noted that multiple residents forgot to complete a task but went back to it while prerounding on the same patient or after prerounding on the other patient. These instances are not reflected in Tables 1 and 2 since residents eventually performed the task.

**Table 2:** Percentage of tasks omitted for at least 1 patient and percentage of residents who completed all tasks by experience level.

Resident experience level	Tasks omitted for at least 1 patient, %	Residents who did not complete all tasks, n/N (%)
PGY^a^-1	24	7/7 (100)
PGY-2	16	5/6 (83)
PGY-3	14	4/7 (57)

^a^PGY: postgraduate year.

### Data Omission by Experience Level

To examine the effect of experience level on the task omission, we calculated the percentage of tasks that were omitted by residents, categorized by their level of experience. Table 2 shows that residents with more experience had lower task omission rates. However, more than half (4/7) of the residents with the most experience (PGY-3) still omitted at least 1 task while prerounding.

Using chi-square tests for independence, we found no significant difference in both the proportion of omitted tasks among experience levels (*χ*^2^_2_=1.8; *P*=.41) and the proportion of residents who did not complete all tasks (*χ*^2^_2_=4.1; *P*=.13).

### Number of Pages Accessed

When responding to the questionnaires prior to participating in the experimental portion of the study, residents cited having to access numerous pages to collect the relevant patient data. Therefore, we wanted to see whether prerounding became more effective and efficient with more experience.

From the video analysis, we noted all pages that were accessed in the EHR when collecting data during prerounding. Pages that were accessed by mistake (ie, mis-clicking on a page then quickly exiting it) or were used mainly to access another page were not included in the analysis since they serve no purpose in data collection. Across all 20 residents, 58 *distinct* pages were accessed while collecting data on the 2 patients during prerounding. Table 3 shows that the total number of distinct pages accessed by each experience group ranged from 35 to 41 pages and did not seem to vary by level of experience.

**Table 3:** Summary of pages accessed to preround 2 patients categorized by experience level.

Years of experience	Aggregate pages visited, n	Average pages visited per participant, n
PGY^a^-1	38	20
PGY-2	35	21
PGY-3	41	21

^a^PGY: postgraduate year.

The mean number of pages accessed by each participant while prerounding was also tabulated. On average, residents accessed 21 pages when prerounding on both patients. Table 3 shows the mean number of pages accessed by residents when categorized by experience level. There was no significant difference in the mean number of pages visited as a function of years in residency (*F*_2,17_=0.08; *P*=.92), suggesting that the mean number of pages visited does not decrease with experience.

### Task Completion Time

We also wanted to see whether EHR system use efficiency improves with experience. While the mean prerounding duration for third-year residents was about 45 seconds faster than first- and second-year residents, it was not statistically significant (*F*_2,19_=0.75; *P*=.48; see Table 4).

**Table 4:** Mean prerounding duration for a patient categorized by experience level.

Years of experience	Mean prerounding duration
PGY^a^-1	6 min 43 s
PGY-2	6 min 43 s
PGY-3	5 min 57 s
Mean across experience levels	6 min 27 s

^a^PGY: postgraduate year.

The video analysis revealed that regardless of experience level, residents spent the most time on the task of reviewing notes. This task was especially time-consuming given residents had to read through the free-form text inputs that varied depending on who inputted the notes.

Another task residents spent a lot of time on was reviewing lab results. The video analysis showed that residents had to frequently scroll both vertically and horizontally during this task, which was noted to be difficult and disorienting based on the residents’ oral comments and questionnaire responses.

### Use of Aggregated Data Pages

From the video analysis, we observed that pages that provided aggregated data for multiple tasks were already implemented within the EHR system. The use of aggregated data pages could potentially reduce the time spent navigating between pages (ie, “Summary/Ped Rounding” page); however, only 3 residents made use of these pages. Of the 3 residents who accessed the aggregated data pages, 2 were in their first year of residency (PGY-1), while 1 was in the second year (PGY-2).

Although the sample size is too small to draw conclusions, it is worth noting that the mean number of pages accessed by the 3 residents was 19 pages, which was slightly lower than the average of 21 pages, but with no statistical significance (t_18_=0.80; *P*=.43, 2-tailed). In contrast to the expectations, residents who used this aggregating page had an average prerounding time of 7:29 minutes, which was higher than the sample average of 6:27, but the difference was not statistically significant as well (t_18_=−1.60; *P*=.12, 2-tailed).

## Discussion

### Principal Findings

The goal of this study was to examine the effect of experience level on EHR use during prerounding. Our study revealed that while most residents reported having enough time and being able to find the information they needed during prerounding, video analysis showed that 80% (16/20) of residents did not complete at least 1 key task. This finding was applicable regardless of experience as over 50% (4/7) of the most experienced residents (PGY-3) still omitted some tasks.

Specifically, our study found that in the specialty focused on in our research (ie, pediatrics acute care wards), the tasks most frequently overlooked were reviewing medications and orders. This finding differs from the results reported in the literature for other specialties. The variations in task omission patterns between our study and those found in the literature suggest that specialty-specific workflow and EHR system design could influence task omission patterns and the quality of pre-rounding. The findings here highlight the importance of identifying workflow-specific solutions that could prevent the omission of tasks and the need for strategies to improve the efficiency of EHR use during prerounding.

### Navigational Challenges in EHR Use

One major challenge residents faced during prerounding was the time spent navigating between pages, which contributed to the inefficiency of the process. On average, residents accessed approximately 21 pages during prerounding, with the number of unique pages accessed amounting to 58 distinct pages. This finding demonstrates an inefficient prerounding process. While summary data pages that consolidate patient data for multiple tasks on a single page were available, most residents chose to gather raw data from different pages instead. It is unclear why residents did not use these summary pages, but it may be due to the lack of training and integration of these pages into the prerounding process or the fact that residents find them confusing or incomplete. This is supported by the fact that residents who did use the summary data pages did not preround any more efficiently than those who did not use them in terms of per-rounding time or number of pages visited.

To improve the efficiency of prerounding, it may be necessary to streamline the process of data collection, such as improving the design and usage of summary pages by tailoring to user needs, providing targeted training on their use, and encouraging residents to use them. EHR providers should also consider other EHR design changes and technological assistance such as artificial intelligence–assistive tools that can facilitate efficient data gathering if summary pages are not providing adequate assistance.

### Specialty and Task Omission

This study revealed a significant variation in data omission rates across tasks, where only labs and meds showed significant omissions among residents. This finding contrasts with a previous study [27], which used the same EHR system to examine omissions among residents in nonpediatric ICU settings that found medication data were almost never omitted (~3%), whereas fluid balance (IOs) was frequently omitted (~37%). Similarly, studies in respiratory ward [37] and general medical ward [38] indicate that fluid balance was often omitted. This disparity suggests that factors such as specialty and care setting may influence data omission rates. For instance, IOs are often more critical to monitor for pediatric than for nonpediatric patients, while medication infusions are more critical in ICU settings than in non-ICU settings, which are supported in the literature. These variations in omission patterns highlight the need to consider contextual factors when designing interventions aimed at improving EHR use efficiency and reducing omission rates.

### Comparison to Prior Work

Our study contributes to the growing body of literature on EHR use in medical settings, specifically regarding prerounding practices in pediatrics as mentioned in the “Specialty and Task Omission” section. Previous studies have shown that there are significant gaps in identifying dangerous medical management issues within EHRs, despite high levels of medical training [30]. These findings are consistent with our own, which revealed that even the most experienced residents still omitted some prerounding subtasks. However, our study adds to the existing literature by specifically examining the completion of prerounding tasks in the context of pediatrics. Furthermore, prior research has also shown that residents often omit collecting some information during prerounding [27]. However, our study expands upon this by revealing that entire tasks were not completed, and more than half of the most experienced residents still omitted some prerounding tasks.

### Recommendations for Improving EHR Use

We believe the lack of improvement in prerounding speed and accuracy with increased experience could be attributed to inadequate EHR training as well as poor EHR design [39]. Based on our findings, interventions to improve the efficiency and effectiveness of prerounding could include checklists within the EHR system or in paper forms to ensure all tasks are completed. Previous work has shown that supporting knowledge in the world versus knowledge in the head—that is, reducing recall and memory—is effective in reducing omission [40]. We recommend the use of checklists that include prompts that remind residents of what information is needed, instead of relying on the residents’ memory each time they preround.

A more comprehensive solution could involve designing the EHR system with case-specific semiautomated workflows for prerounding, which would suggest relevant pages to residents that can help them complete the required tasks. This would ensure that each prerounding task is not only completed but also done in the intended manner. This would necessitate the need to conduct a hierarchical task analysis [41] to decompose the overall prerounding task into goals, subgoals, operations, and plans to determine how the EHR could best support the residents at each level.

Studies have shown that the use of automatically generated templates had a positive impact on residents’ performance during rounding, including omission rates [35,42,43]; however, the use of such automation techniques could impact the residents’ situational awareness and cause overreliance on the automation [44]. Therefore, the impact of introducing artificial intelligence automations should be studied more before implementing them within EHR systems.

Furthermore, we recommend implementing training programs for residents that are tailored for specific tasks such as prerounding to standardize the process and introduce the residents to system features that might be useful and time-saving when prerounding. For example, training programs could recommend structured sequential procedures for completing tasks and introduce residents to the different functionalities of pages and new dashboards that allow for faster and more centralized information access [45]. Such training programs could be implemented as training sessions, system walkthroughs, or web-based videos that are accessible when needed [46]. However, the efficacy of the training program and its added work burden on the residents should be considered before implementation.

The design of the EHR system should also be reconsidered to better support the work of the residents [36]. Information access cost should be reduced, and features should be made clearly visible to users in ways that eliminate the need for training, and instead, users can explore system features on their own.

### Strengths and Limitations

This study has several notable strengths that contribute to the understanding of EHR use during the prerounding process. First, our mixed methods approach, which combines self-reported data with video analysis, is allowing for a comparison between residents’ perceived performance and their actual performance and is enabling a more accurate evaluation of EHR use.

Second, the focus on the pediatric specialty provides valuable insights into the unique challenges faced by pediatricians and allows comparison of the EHR usage patterns to other specialties studied in the literature. Third, the varying experience levels among participants allow for a broader perspective on the impact of experience on EHR usage and performance.

However, this study is not without limitations. First, the study was limited to a single setting, a single medical center, one department, and using a single EHR system, which may limit the generalizability of our findings, and additionally, the use of EHR for prerounding may have unique considerations for pediatricians when compared to other specialties. Second, the small sample size of this study may have limited the statistical power of our analyses. However, the combination of data collected was among the few of its kind, and we performed time-intensive analyses that revealed new trends and supported existing work. We also acknowledge the need for caution in generalizing our results due to the majority of the residents being females, which may have introduced potential gender bias into our findings.

### Future Work

For future work, building on the strengths of our study, larger-scale studies across multiple settings and specialties could be conducted to confirm the generalizability of our findings. This would help to establish the validity of our conclusions and allow for broader insights into EHR use during prerounding across different clinical contexts.

Moreover, given the identified tasks that were frequently omitted, future research could focus on exploring the underlying reasons behind this discrepancy. Specifically, research could study how different clinical roles or specialties may affect task omission rates and how interventions such as checklists and workflow automations could be tailored to address these differences.

### Conclusions

Overall, our findings reveal that residents often omitted completing tasks while prerounding and the process was largely inefficient due to the EHR design, lack of proper training, and an unstandardized prerounding process. To improve EHR use efficiency and prevent omissions, interventions such as checklists, training programs, and customized EHR interfaces are suggested. Despite its limitations, our study provides important insights about specialty-specific EHR challenges and those associated with EHR use during prerounding in general.

## Appendix

Multimedia Appendix 1 Questionnaires questions.

Multimedia Appendix 2 Patient cases descriptions.

Multimedia Appendix 3 Spreadsheet format.

## Abbreviations

EHR: electronic health record
ICU: intensive care unit
IO: intake and output
PGY: postgraduate year
STROBE: Strengthening the Reporting of Observational Studies in Epidemiology

## Data Availability

The data that support the findings of this study are available from the corresponding author upon reasonable request and with approval from the institutional review board.
