# Supplementary material for: Examining Pediatric Resident Electronic Health Records Use During Prerounding: Mixed Methods Observational Study
Source: JMIR Med Educ. 2023 May 10;9:e38079. doi: 10.2196/38079 (PMC10209786; doi:10.2196/38079)
Supplement: Multimedia Appendix 1 [file mededu_v9i1e38079_app1.docx]

### Online Questionnaire Questions

1. What is your level of training?
   1. PYG-1
   2. PYG-2
   3. PYG-3
2. On average, how much time each day do you allocate to pre-round in the mornings when you are working on the acute care wards?
   1. less than 15 minutes
   2. between 15 and 30 minutes
   3. between 30 and 45 minutes
   4. between 45 and 60 minutes
   5. more than 60 minutes
3. On average, how many minutes do you think you spend pre-rounding in [EHR Software] on an individual patient on the acute care wards?
   1. less than 5 minutes
   2. between 5 and 10 minutes
   3. between 10 and 15 minutes
   4. more than 15 minutes
4. When pre-rounding [in the department], are there particular things you find hard to find?
   1. Yes
   2. No

If yes, what do you find hard to find? (Free form answer)

1. When pre-rounding on the acute care wards, do you write down the information you collect?
   1. Yes
   2. No

If yes, how/where do you record it? (Free form answer)

1. What do you find most frustrating about the pre-rounding process [in the department]?
2. How could we make the pre-rounding process [in the department] more efficient and more effective? (Free form answers)

### Beginning of Experiment Questionnaire

1. What is your level of training?
   1. PGY-1
   2. PGY-2
   3. PGY-3
2. How much time have you spent [In the department]?
   1. <1 month
   2. 1 month
   3. 2 months
   4. 3 months
   5. >3 months
3. How efficient do you think you are at pre-rounding [In the department]? (1-5 scale)
4. How often do you find all the information you are looking for when pre-rounding? (1-5 scale)
5. Is there certain information you frequently have trouble finding and if so what is it?

### End of Experiment Questionnaire

1. Did you have enough time to complete your pre-rounding?
   1. Yes
   2. No
2. Did you find all the information you were looking for?
   1. Yes
   2. No
3. If there was information you couldn't find, what was it?
