# Supplementary material for: Examining Pediatric Resident Electronic Health Records Use During Prerounding: Mixed Methods Observational Study
Source: JMIR Med Educ. 2023 May 10;9:e38079. doi: 10.2196/38079 (PMC10209786; doi:10.2196/38079)
Supplement: Multimedia Appendix 2 [file mededu_v9i1e38079_app2.docx]

Both cases were of moderate complexity and representative of typical patients admitted to the acute care wards in which the residents work:

1. Long term hospitalization for a young infant who was born moderately preterm with a complex congenital heart disease. The patient was being fed expressed break milk via nasogastric tube due to oral aversion and had intermittent regurgitation but was growing well and had no respiratory difficulties. The patient was expected to have a definitive surgical repair of her congenital heart disease in the near future.
2. Day three of hospitalization for a male patient in his middle childhood with systemic lupus erythematosus and lupus nephritis admitted with hypertension and worsening renal function and anemia. The patient was being treated with a mix of medications, fluid resuscitation, and blood transfusion.
