# Supplementary material for: Examining Pediatric Resident Electronic Health Records Use During Prerounding: Mixed Methods Observational Study
Source: JMIR Med Educ. 2023 May 10;9:e38079. doi: 10.2196/38079 (PMC10209786; doi:10.2196/38079)
Supplement: Multimedia Appendix 3 [file mededu_v9i1e38079_app3.docx]

#### Fields

- Timestamp: time stamp within the video at which an event occurred (for future reference and for computations)
- Entry Type:
  - Pre-rounding start/end: denotes the start or end of pre-rounding on a patient.
  - Subtask start/end: denotes the start or end of one of the six subtasks.
  - Page access: denotes a page access event.
  - Data collection: denotes a data collection event by the participant. (was not used in the analysis)
  - Comment: a comment from the reviewer or from the participant (specified within the designated field).
- Patient number: The patient to which the event is related to.
- Task: the subtask that the participant is performing.
- Chart viewed: the page which the participant is currently on.
- Data Collected: What data was being collected. (Not used in the analysis)
- Participant comment: a comment that is made by the participant during the experiment.
- Reviewer comment: a comment by the reviewer who is reviewing the recording.
- Video number: a unique identifier for each recording. (Auto populated)
- Years of experience: participant’s years of experience. (Auto populated)

#### Screenshot


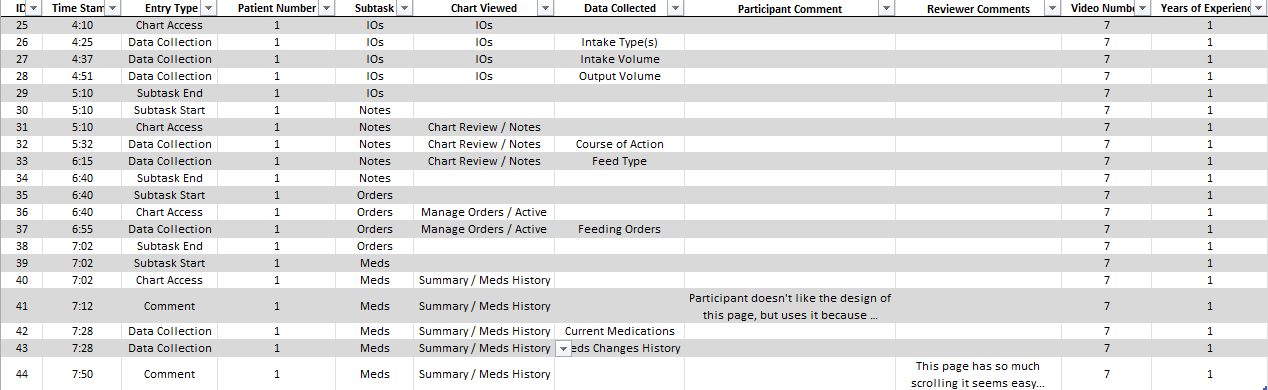


#### Notes

The Excel sheet was designed inhouse and embedded with code to standardize the data entry between reviewers. Entry Type, patient Number, Subtask, Chart Viewed, and Data Collected fields were bound to predefined dropdown lists. The reviewer had to either select the entry from the dropdown list which contained a predefined list of options, or choose to add a new entry to the list through a prompt (used of Chart Viewed and Data Collected fields only). The list of options was continuously synced across reviewers through secure data hosting.

This helped in standardizing the entries across reviewers as the prompt would show them a list of similar options before allowing them to add a new one.
